# Superb microvascular imaging for distinguishing thyroid nodules: A meta-analysis (PRISMA): Retraction

**DOI:** 10.1097/MD.0000000000042734

**Published:** 2025-06-06

The article, “Superb microvascular imaging for distinguishing thyroid nodules: A meta-analysis (PRISMA)”,^1^ which appears in Volume 101, Issue 24 of *Medicine*, is being retracted over concerns with the methodology. After publication, concerns were raised over references to the “Begger’s test,” which does not exist in the literature and is indicative of possible fraud. Upon further investigation, authorship irregularities were also discovered in our submission system. As a result, the Publisher no longer has confidence in the integrity of the content or the authorship.
